# Model difference in the effect of cilostazol on the development of experimental pulmonary hypertension in rats

**DOI:** 10.1186/s12890-021-01710-4

**Published:** 2021-11-20

**Authors:** Toshikazu Ito, Erquan Zhang, Ayaka Omori, Jane Kabwe, Masako Kawai, Junko Maruyama, Amphone Okada, Ayumu Yokochi, Hirofumi Sawada, Yoshihide Mitani, Kazuo Maruyama

**Affiliations:** 1 Department of Anesthesiology and Critical Care Medicine, Mie University Graduate School of Medicine, 2-174 Edobashi, Tsu, Mie 514-8507 Japan; 2 Fuzhou Children’s Hospital of Fujian Province Affiliated with Fujian Medical University, 145-817-Middle Road, Gulou, Fuzhou, 350005 Fujian China; 3 Faculty of Health Science, Suzuka University of Medical Science, Suzuka, Mie 510-0293 Japan; 4 Department of Pediatrics, Mie University Graduate School of Medicine, 2-174 Edobashi, Tsu, Mie 514-8507 Japan

**Keywords:** Monocrotaline, Chronic hypoxia, Cilostazol, Pulmonary hypertension, Nitric oxide

## Abstract

**Background:**

Preventing pulmonary vascular remodeling is a key strategy for pulmonary hypertension (PH). Causes of PH include pulmonary vasoconstriction and inflammation. This study aimed to determine whether cilostazol (CLZ), a phosphodiesterase-3 inhibitor, prevents monocrotaline (MCT)- and chronic hypoxia (CH)-induced PH development in rats.

**Methods:**

Fifty-one male Sprague–Dawley rats were fed rat chow with (0.3% CLZ) or without CLZ for 21 days after a single injection of MCT (60 mg/kg) or saline. Forty-eight rats were fed rat chow with and without CLZ for 14 days under ambient or hypobaric (air at 380 mmHg) CH exposure. The mean pulmonary artery pressure (mPAP), the right ventricle weight-to-left ventricle + septum weight ratio (RV/LV + S), percentages of muscularized peripheral pulmonary arteries (%Muscularization) and medial wall thickness of small muscular arteries (%MWT) were assessed. Levels of the endothelial nitric oxide synthase (eNOS), phosphorylated eNOS (peNOS), AKT, pAKT and IκB proteins in lung tissue were measured using Western blotting. Monocyte chemotactic protein (MCP)-1 mRNA in lung tissue was also assessed.

**Results:**

mPAP [35.1 ± 1.7 mmHg (MCT) (n = 9) vs. 16.6 ± 0.7 (control) (n = 9) (*P* < 0.05); 29.1 ± 1.5 mmHg (CH) (n = 10) vs. 17.5 ± 0.5 (control) (n = 10) (*P* < 0.05)], RV/LV + S [0.40 ± 0.01 (MCT) (n = 18) vs. 0.24 ± 0.01 (control) (n = 10) (*P* < 0.05); 0.41 ± 0.03 (CH) (n = 13) vs. 0.27 ± 0.06 (control) (n = 10) (*P* < 0.05)], and %Muscularization and %MWT were increased by MCT injection and CH exposure. CLZ significantly attenuated these changes in the MCT model [mPAP 25.1 ± 1.1 mmHg (n = 11) (*P* < 0.05), RV/LV + S 0.30 ± 0.01 (n = 14) (*P* < 0.05)]. In contrast, these CLZ effects were not observed in the CH model. Lung eNOS protein expression was unchanged in the MCT model and increased in the CH model. Lung protein expression of AKT, phosphorylated AKT, and IκB was downregulated by MCT, which was attenuated by CLZ; the CH model did not change these proteins. Lung MCP-1 mRNA levels were increased in MCT rats but not CH rats.

**Conclusions:**

We found model differences in the effect of CLZ on PH development. CLZ might exert a preventive effect on PH development in an inflammatory PH model but not in a vascular structural change model of PH preceded by vasoconstriction. Thus, the preventive effect of CLZ on PH development might depend on the PH etiology.

## Background

Pulmonary hypertension (PH) is characterized by an increase in pulmonary artery pressure (PAP), right ventricular hypertrophy (RVH), and functional and/or structural vascular changes [1, 2]. Possible causes of PH include pulmonary vasoconstriction, diffuse microthromboembolism, and pulmonary vascular remodeling [1–3]. In all conditions causing PH in humans [3–5] and experimental models [6–20], vascular changes include new muscularization of normally nonmuscular peripheral pulmonary arteries and medial hypertrophy of muscular arteries. PH may be encountered in the intensive care unit in patients with acute respiratory distress syndrome (ARDS) [21–23], congenital heart disease with left-to-right shunt [3, 24, 25], mitral valve disease [26], and interstitial pulmonary fibrosis [27], as well as after cardiothoracic surgery [28, 29]. Nitric oxide (NO) is a vasodilator and suppressor of smooth muscle cell proliferation [1, 2], and the bioavailability of NO is reduced in patients with PH and in experimental PH models [30–32]. We and other researchers have shown that modulators that increase NO production ameliorate the development of PH and vascular remodeling [6, 7, 33].

Cilostazol (CLZ) is a selective phosphodiesterase-3 inhibitor that increases intracellular cyclic AMP levels, which inhibits platelet aggregation and induces peripheral vasodilation. The antiplatelet agent CLZ is indicated for intermittent claudication in patients with peripheral arterial disease [34], thrombotic complications of coronary angioplasty [35] and secondary stroke prevention [36]. Through cAMP-dependent and cAMP-independent mechanisms, CLZ also induces the phosphorylation of endothelial nitric oxide synthase (eNOS), which increases NO production in the aortas of diabetic rats [37], human aortic endothelial cells [38], and rat cultured smooth muscle cells [39]. CLZ also exerts anti-inflammatory effects [40–42]. By activating the NO synthase-NO pathway or preventing inflammatory responses, CLZ might prevent the development of PH, as suggested in an earlier study [43] in which PH was not fatal. Since the pathogenesis and severity of PH are heterogeneous [1–3, 44], we determined the effect of CLZ on the development of two established experimental models of PH: rat models of MCT-induced PH [6, 8, 12–15, 17, 18, 33, 43, 45–50] and chronic hypoxia (CH)-induced PH [6, 7, 9–11, 19, 20, 31, 32, 51]. The rats with MCT-induced PH used in this study were a model of experimental fatal PH.

## Methods

The Animal Experiment Committee of Mie University School of Medicine approved the study protocol (Nos. 20-34 and 20-35). Rats were fed rat chow containing 0.3% CLZ [52] or control chow without CLZ. Rat chow with and without CLZ was a gift from Otsuka Pharmaceutical Co., Ltd (Tokushima, Japan). Sprague–Dawley rats were obtained from Japan SLC, Inc. In total 159 rats were used. For euthanasia rats were administered 50 mg/kg pentobarbital sodium (Somnopentil®, Kyorituseiyaku Corporation, Japan) via intraperitoneal injection. After obtaining no consciousness with respiratory depression and no response to the stimulation, which took about 5–10 min and showed deep anesthesia, rats were put under mechanical ventilation through tracheostomy. Then the abdomen was incised and the rats were exsanguinated by aortic incision and heart and lung samples were removed.

### Animal groups

#### MCT21 model

**Fig. 1:**
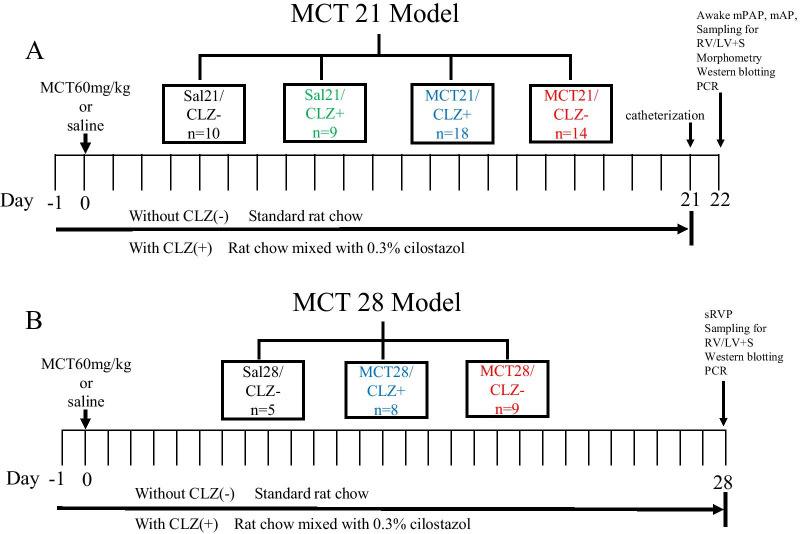
Experimental protocol used to establish the MCT21 and MCT28 models. **A**, MCT21 model: pulmonary and carotid arteries were catheterized 21 days after the MCT or saline injection; awake mean pulmonary artery pressure (mPAP) and mean artery pressure (mAP) were measured at day 22 when rats were fully awake; lung and heart samples were obtained for measurements of the right ventricle (RV) weight-to-left ventricle + septum (LV + S) weight ratio (RV/LV + S), morphometry of pulmonary arteries, Western blotting and PCR after the pressure measurements. **B**, MCT28 model: measurement of systolic right ventricular pressure (sRVP) under anesthesia and sampling for RV/LV + S, Western blotting and PCR were performed 28 days after the injection of MCT or saline. Sal/CLZ-, rats injected with saline and fed rat chow without cilostazol (CLZ); Sal/CLZ +, rats injected with saline and fed rat chow with CLZ; MCT/CLZ-, rats injected with MCT and fed rat chow without CLZ; MCT/CLZ +, rats injected with MCT and fed rat chow with CLZ. MCT, monocrotaline; Sal, saline; n =, number of rats used. We were not always successful in obtaining all these datasets or samples for each assigned rat because of technical reasons, especially when recording mPAP, and thus the number of rats used (n) was not always the same as the numbers listed in Figs. 5, 6, and 9

Seven-week-old male Sprague–Dawley rats (SLC, Japan) weighing 185–245 g were used. Rats were fed rat chow with or without CLZ one day before the administration of a single injection of MCT (60 mg/kg, Sigma) or saline and continued to be fed the same rat chow for another 21 days (Fig. 1A). Each animal was randomly assigned to one of four groups: (1) a single injection of saline and rat chow without CLZ (Sal21/CLZ-) (n = 10), (2) a single injection of saline and rat chow with CLZ (Sal21/CLZ +) (n = 9), (3) a single injection of MCT and rat chow without CLZ (MCT21/CLZ-) (n = 18), and 4) a single injection of MCT and rat chow with CLZ (MCT21/CLZ +) (n = 14). MCT (60 mg/kg) [12–15, 45, 48, 50] or the same volume of 0.9% NaCl was subcutaneously injected into the hind flank.

#### CH model

**Fig. 2:**
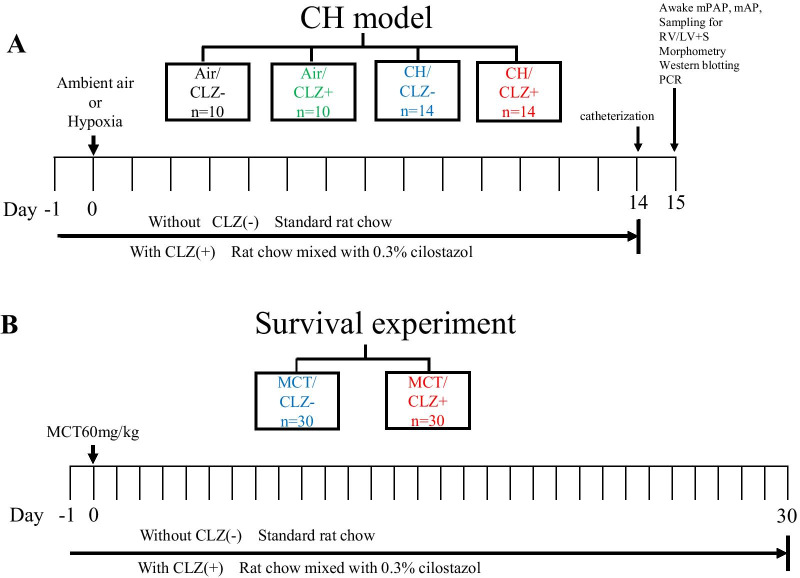
Experimental protocol used to establish the CH model and survival study. **A**, CH model: Pulmonary and carotid arteries were catheterized on the final day of chronic hypoxia (CH) exposure; awake mean pulmonary artery pressure (mPAP) and mean artery pressure (mAP) were measured on day 15 with rats fully awake; lung and heart samples were obtained for measurements of the right ventricle weight-to-left ventricle + septum weight ratio (RV/LV + S), morphometry of pulmonary arteries, Western blotting and PCR after the pressure measurements. Air/CLZ-, rats exposed to ambient air and fed rat chow without CLZ; Air/CLZ +, rats exposed to ambient air and fed rat chow with CLZ; CH/CLZ-, rats exposed to chronic hypoxia and fed rat chow without CLZ; CH/CLZ +, rats exposed to chronic hypoxia and fed rat chow with CLZ. (n) = number of rats used. The number of rats used (n) was not always the same as the numbers listed in Figs. 5, 6 and 10. See the legend of Fig. 1. **B**, Survival experiment. MCT/CLZ-, rats injected with MCT and fed rat chow without CLZ. MCT/CLZ +, rats injected with MCT and fed rat chow with CLZ. MCT, monocrotaline; (n) = number of rats used

Seven-week-old male Sprague–Dawley rats (SLC, Japan) weighing 187–235 g were used. Rats were fed rat chow with or without CLZ beginning one day before the start of hypobaric CH exposure (air at 380 mmHg) and continued to be fed the same rat chow until the final day of ambient air or CH exposure (Fig. 2A). Each animal was randomly assigned to one of four groups: (1) rats exposed to ambient air without CLZ (Air/CLZ-) (n = 10), (2) rats exposed to ambient air with CLZ, (Air/CLZ +) (n = 10), (3) rats exposed to CH without CLZ (CH/CLZ-) (n = 14) and (4) rats exposed to CH with CLZ (CH/CLZ +) (n = 14). Rats were exposed to hypoxia for 14 days and returned to ambient air after catheterization [6, 9–11].

#### MCT28 model

We performed a limited study with 3 groups (Sal28/CLZ-, MCT28/CLZ-, and MCT28/CLZ +) to determine whether the observations recorded in rats for 21 days would persist for 28 days. In the MCT28 model, each animal injected with saline or MCT (60 mg/kg) was randomly assigned to one of three groups (Fig. 1B): Sal28/CLZ- (n = 5), MCT28/CLZ- (n = 8), and MCT28/CLZ + (n = 9). Rats were fed for 28 days after the injection of MCT as in the MCT21 model. The MCT28 model rats were used to evaluate systolic right ventricular pressure (sRVP) under 45 mg/kg pentobarbital anesthesia and RVH and to obtain lung samples for protein and mRNA assays (Fig. 1B). The MCT21 and CH model rats were used to evaluate awake mean PAP (mPAP), right ventricle weight-to-left ventricle + septum weight ratio (RV/LV + S), and pulmonary vascular structural changes and to obtain lung samples for protein and mRNA assays (Figs. 1A, 2A).

### mPAP and mean artery pressure (mAP) in MCT21 and CH, and sRVP in MCT28

At the end of 21 days after the MCT injection and 14 days of CH exposure, a pulmonary artery catheter (silastic tubing, 0.31 mm ID and 0.64 mm OD) was inserted into rats through the right external jugular vein into the pulmonary artery employing a closed-chest technique under 45 mg/kg pentobarbital anesthesia with no tail movement upon stimulation [10, 11, 13] (Figs. 1A, 2A). The left internal carotid artery was also cannulated. Twenty-four hours after the catheterization, the mPAP and mAP were recorded in fully conscious rats with a physiological transducer and an amplifier system (AP 620G, Nihon Kohden, Japan) once the rats were calm (Figs. 1A, 2A).

In the MCT28 model, at the end of 28 days after MCT injection, sRVP was measured in rats under 45 mg/kg pentobarbital anesthesia using the closed-chest technique, and then lung samples for protein and mRNA assays were obtained (Fig. 1B).

### Preparation of lung tissue for morphometric analysis and lung tissue sampling for protein and mRNA assays

After the measurement of awake mPAP in the MCT21 and CH models, the rats were anesthetized with 50 mg/kg pentobarbital again and mechanically ventilated through tracheostomy. The abdomen was then incised, and the abdominal aorta was incised to cause blood loss and euthanasia. A midline sternotomy was performed to expose the heart and lung. The hilum of the right lung was ligated, and the right lung was excised and placed in liquid nitrogen for real-time polymerase chain reaction (PCR) and Western blotting of whole lung tissue. Blood samples were collected for the hematocrit measurement. Sections of the left lung were prepared for a morphometric analysis of the vasculature using the barium injection method [6, 9–14] to identify peripheral pulmonary arteries. Briefly, the left pulmonary artery was injected with a hot radiopaque barium-gelatin mixture at 100 cm H_2_O pressure [6, 9–14]. After injection, the lung was distended and perfused through the tracheal tube with 10% formalin at 36 cm H_2_O pressure for 72 h. Sections were stained for elastin using the Van Gieson method. The right ventricle (RV) of the heart was dissected from the left ventricle plus septum (LV + S) and weighed separately. The heart weight ratio (RV/LV + S) was calculated to assess RVH. We also evaluated fibrosis in the right ventricle by performing Masson’s trichrome staining for collagen in 3 randomly selected heart tissue samples from each group (Air/CLZ-(control), MCT21/CLZ-, MCT21/CLZ +, CH/CLZ-, and CH/CLZ +). Lung sampling from the MCT28 group is described above.

### Morphometric analysis of pulmonary arteries

Sections were analyzed under a light microscope by an investigator without previous knowledge of the treatment groups. All barium-filled arteries in each tissue section were examined at × 400 magnification, for an average of 220 arteries per section (110–340 arteries per section). Each artery was identified as being one of two structural types to determine the presence of muscularity: muscularized (with a complete medial coat, incomplete medial coat, or only a crescent of muscle being present) and nonmuscular (no muscle apparent) [6, 9–14]. The percentages of muscularized arteries (%Muscularization) in peripheral pulmonary arteries with an external diameter between 15 and 50 µm and those between 51 and 100 µm were calculated. For muscular arteries between 101 and 200 µm in diameter (an average of 18 arteries (5–20 arteries) were observed in each section), the wall thickness of the media (distance between external and internal elastic laminae) was measured along the shortest curvature, and the percent medial wall thickness (%MWT), the ratio of the wall thickness of the media to the external diameter in muscular arteries, was calculated [6, 9–14].

### Western blotting for eNOS, peNOS, AKT, pAKT, IκB, and HMGB-1

For Western blotting, lung samples were randomly selected from the MCT21, MCT28, and CH models, where all pooled lung samples were unable to be used because of the number of gel lanes. Samples were homogenized, and the supernatant was standardized to a concentration of 3.0 mg/ml. Thirty micrograms of total protein from each sample were subjected to SDS-PAGE on 10% polyacrylamide gels (Nacalai, Japan) and blotted onto a PVDF membrane (Amersham Hybond-P, GE Healthcare). Blots were blocked for 1 h with 5% skim milk diluted in 0.1% TBST (Tris-buffered saline plus Tween) followed by an overnight incubation at 4 °C with a primary antibody diluted in Can Get Signal Immunoreaction Enhancer Solution 1 (Toyobo Co. Ltd., Japan). Six different primary antibodies against the following proteins were used: endothelial nitric oxide synthase (eNOS) (BD Transduction Laboratories; G10296, lot 21527, 1:4000 dilution), phosphorylated eNOS (peNOS) (Cell Signaling; phospho-eNOS, ser-1177 #9751, 1:2000 dilution), serine-threonine protein kinase (AKT) (Cell Signaling; #4814S, 1:2000 dilution), phosphorylated AKT (pAKT) (Cell Signaling; #460P, 1:2000 dilution), IκB-α (Cell Signaling; #4814S, 1:2000 dilution), high-mobility group box-1 (HMGB-1) (Cell Signaling; #3935S, 1:2000 dilution), and β-actin (Sigma; A5441, 1:200,000 dilution). Next, the blots were incubated with the secondary antibody (Amersham NA 931, 1:20,000 dilution) diluted in Can Get Signal Immunoreaction Enhancer Solution 2 (Toyobo Co. Ltd., Japan) for 1 h at room temperature and in Immobilon Western Chemiluminescent HRP Substrate (Millipore Corporation, USA) for 5 min. Luminescent signals were captured digitally, and densitometry was performed using Multi Gauge Ver. 3.0 (Fujifilm, Science Laboratory 2005, Japan). Each target protein was normalized to β-actin, and the relative fold change compared to the control group (100%) was calculated.

### cDNA preparation and PCR

Levels of the eNOS, AKT, and monocyte chemotactic protein-1 (MCP-1) mRNAs in whole lung tissue were determined using real-time PCR. After the extraction of total RNA from whole lung tissue using TRIzol reagent (Invitrogen, USA), cDNA synthesis was performed with ReverTra Ace (Toyobo Co., Ltd., Biochemical Operations Department, Osaka, Japan). The cDNA templates (15 ng of total RNA) were amplified with a StepOne Plus Real Time PCR System (Applied Biosystems). The sequences of the primer pairs are listed in Table 1. Relative quantification was performed with the comparative ∆∆Ct method by normalization to the β-actin mRNA.

**Table1:** Primer list

Gene name	Primer (5′–3′) sequence
eNOS	F: ATGGATGAGCCAACTCAAGG
	R: GGCTGCAGTCCTTTGATCTC
AKT	F: GGTCGTGGGTCTGGAATG
	R: AGAAGGAGGTCATCGTTGC
MCP-1	F:TGAACTTGACCCATAAATCTGAAG
	R:AAGGCATCACATTCCAAATCAC
ACTB	F: GACGGTCAGGTCATCACTATCG
	R: TAG TTTCATGGATGCCACAGGAT

eNOS, Endothelial nitric oxide synthase; AKT, Serine-threonine kinase; MCP-1, Monocyte chemotactic protein-1; ACTB, β-actin

### Survival experiment

Sixty rats (7-week-old male Sprague–Dawley rats (SLC, Japan)) were used. Each rat was randomly assigned to one of two groups: 30 rats (weighing 213–234 g) fed rat chow without CLZ and 30 rats (195–233 g) fed rat chow with CLZ (Fig. 2B). The rat chow with CLZ (including 0.3% CLZ) was the same as that provided to establish the MCT21, MCT28, and CH models. One day after the assignment of the feeding group, all rats were subcutaneously injected with MCT (60 mg/kg). Food and water were provided ad libitum. The number of living rats was counted daily, and the Kaplan–Meier survival curve was constructed for up to 30 days after the injection of MCT.

### Data analysis

Values are presented as the means ± SE. When more than two means were compared, one-way analysis of variance was used. When significant variance was found, Fisher’s protected least significant difference test was employed to establish which groups were different. Survival was evaluated using the Breslow-Gehan-Wilcoxon test in StatView5.0 software. Differences were considered significant at *P* < 0.05.

## Results

### Body weight

#### MCT21 model

**Fig. 3:**
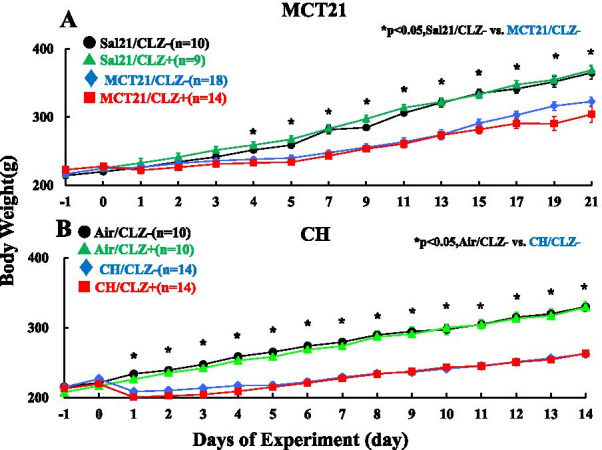
Body weight. **A** Rats injected with monocrotaline (MCT). Sal21/CLZ-, rats injected with saline and fed rat chow without cilostazol (CLZ); Sal21/CLZ +, rats injected with saline and fed rat chow with CLZ; MCT21/CLZ-, rats injected with MCT and fed rat chow without CLZ; MCT21/CLZ +, rats injected with MCT and fed rat chow with CLZ. (n) = number of rats, means ± SE. ✱ *P* < 0.05, comparison of the Sal/CLZ- group and the MCT/CLZ- group. **B** Rats exposed to chronic hypoxia (CH). Air/CLZ-, rats exposed to ambient air and fed rat chow without CLZ; Air/CLZ +, rats exposed to ambient air and fed rat chow with CLZ; CH/CLZ-, rats exposed to chronic hypoxia and fed rat chow without CLZ; CH/CLZ +, rats exposed to chronic hypoxia and fed rat chow with CLZ. (n) = number of rats, means ± SE. ✱ *P* < 0.05, comparison of the air/CLZ- group and the CH/CLZ- group

All rats gained body weight steadily. MCT rats had significantly lower body weights than saline control rats from day 4 to the last day of the experiment, probably due to the lower amount of food intake. A previous study, where daily food intake was measured, reported less food intake in MCT rats than in control rats (12), which showed that MCT rats might have less of an appetite, and our present results are at least partially consistent with this observation. CLZ had no effects on body weight gain in either the MCT or saline control rats (Fig. 3A).

#### CH model

CH-exposed rats lost weight during the first several days of hypoxia exposure but regained weight afterward. The air-exposed rats gained weight steadily. After the start of hypoxia exposure, CH-exposed rats showed significantly lower body weights than air-exposed rats. CLZ treatment had no effect on the body weight of either air- or CH-exposed rats (Fig. 3B).

### Dosage of CLZ

#### MCT model

**Fig. 4:**
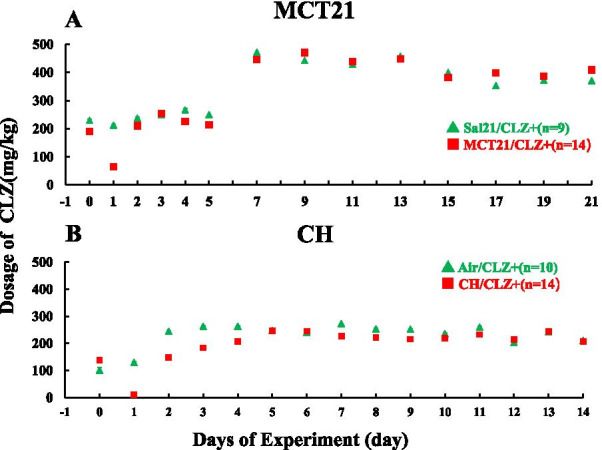
Dosage of CLZ calculated based on food intake. **A**, Rats injected with monocrotaline (MCT). Each plot is the average dosage per kg per day (from day 0 to day 5) or per 2 days (from day 7 to day 21) for a rat. For example, the plot of day 0 is the dosage a rat had consumed for 24 h from day -1 to day 0, and the plot of day 21 is the dosage a rat had consumed for 48 h from day 19 to day 21. Sal21/CLZ +, rats injected with saline and fed rat chow with CLZ; MCT21/CLZ +, rats injected with MCT and fed rat chow with CLZ. **B**, Rats exposed to chronic hypoxia (CH). Each plot is the average dosage per kg per day. For example, the plot of day 0 is the dosage a rat had consumed for 24 h from day -1 to day 0, and the plot of day 14 is the dosage a rat had consumed for 24 h from day 13 to day 14. Air/CLZ +, rats exposed to ambient air and fed rat chow with CLZ; CH/CLZ +, rats exposed to chronic hypoxia and fed rat chow with CLZ

Approximate dosages (mg) of CLZ were calculated using the equation: food intake (g) × 0.003 × 1000, which is the average dosage per kg per day throughout the experimental course. The dosage of CLZ was ~ 230 mg/kg/day in the Sal/CLZ + group and ~ 200 mg/kg/day in the MCT/CLZ + group (Fig. 4A). Although the dosage decreased to 64 mg/kg/day on the day of MCT injection, the dosage was similar in the Sal/CLZ + and MCT/CLZ + groups beginning on day 4 and thereafter throughout the experiment.

#### CH model

The dosage of CLZ was ~ 250 mg/kg/day in the Air/CLZ + group and ~ 220 mg/kg/day in the CH/CLZ + group (Fig. 4B). Although the dosage decreased to 11 mg/kg/day on the first day of hypoxia exposure, the dosage was similar in the Air/CLZ + and CH/CLZ + groups from day 4 throughout the experiment.

### mPAP, mPAP/mAP, RV/LV + S, mAP and sRVP

#### MCT21 model

**Fig. 5:**
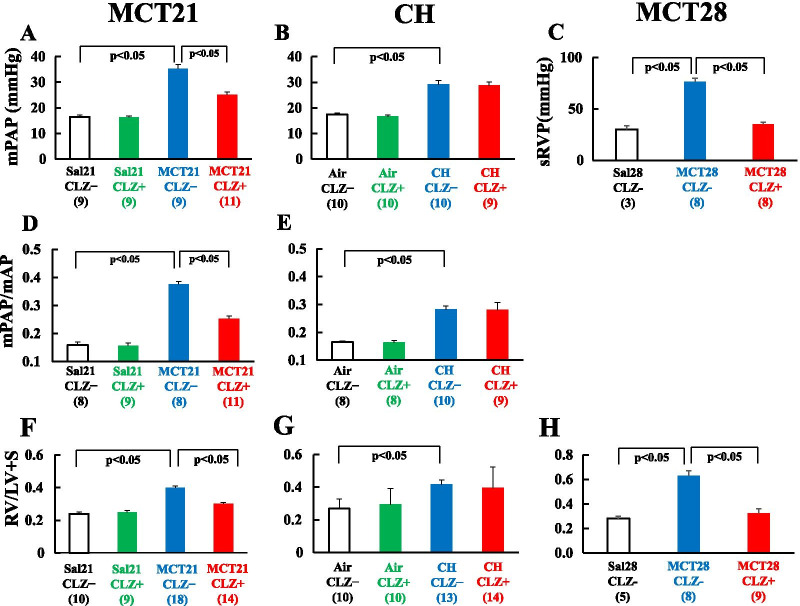
mPAP, mPAP/mAP, RV/LV + S, and sRVP. The left column (MCT21) shows rats in the MCT21 group. The middle column (CH) shows rats in the CH group (rats exposed to chronic hypoxia for 14 days). The right column (MCT28) represents rats in the MCT28 group. **A**, **B**: Mean pulmonary artery pressure (mPAP); **C**: Systolic right ventricular pressure (sRVP); **D**, **E**: Ratio of mPAP to mean artery pressure (mPAP/mAP); **F–H**: Right ventricle (RV) weight-to-left ventricle + septum (LV + S) weight ratio (RV/LV + S), sRVP under anesthesia, and RV/LV + S were measured in rats 28 days after the injection of monocrotaline (MCT28). We measured systolic right ventricular pressure (sRVP) in MCT28 rats under anesthesia instead of mPAP, because obtaining mPAP is technically difficult. (n) = number of rats, means ± SE. The number in parentheses is the number of rats in which mPAP was successfully measured

**Fig. 6:**
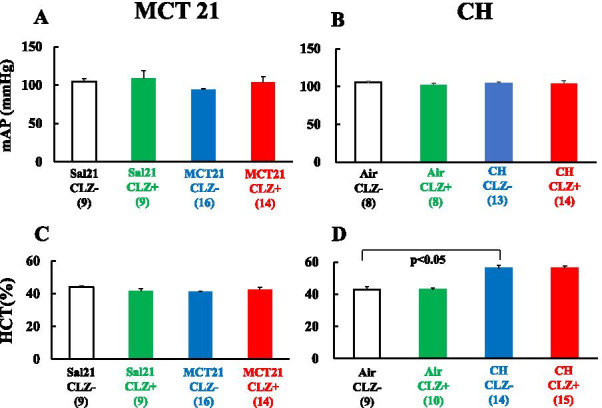
Mean artery pressure (mAP), and hematocrit (HCT). The left column (MCT21) represents in rats in the MCT21 group. The right column (CH) shows rats in the CH group (rats exposed to chronic hypoxia for 14 days). **A**: Mean artery pressure in the MCT21 group; **B**: Mean artery pressure in the CH group; **C**: Hematocrit (HCT) in the MCT21 group; **D**: Hematocrit (HCT) in the CH group. Sal, injected with saline; MCT, injected with monocrotaline (MCT); CLZ-, fed rat chow without CLZ; CLZ +, fed rat chow with CLZ. (n) = number of rats, means ± SE

When comparing the effects of MCT administration between the Sal21/CLZ- and MCT21/CLZ- groups, mPAP [16.56 ± 0.73 (n = 9) vs. 35.33 ± 1.67 (n = 9) mmHg (*P* < 0.05) (Fig. 5A)], mPAP/mAP [0.16 ± 0.01 (n = 8) vs. 0.38 ± 0.01 (n = 8) (*P* < 0.05) (Fig. 5D)], and RV/LV + S [0.24 ± 0.01 (n = 10) vs. 0.40 ± 0.01 (n = 18) (*P* < 0.05) (Fig. 5F)] were all significantly higher in the MCT21/CLZ- group, suggesting that MCT caused PH and RVH. In the comparison of the effects of CLZ treatment between the MCT21/CLZ + and MCT21/CLZ- groups, mPAP [25.09 ± 1.06 mmHg (n = 11) (*P* < 0.05)], mPAP/mAP [0.25 ± 0.01 (n = 11)], and RV/LV + S [0.30 ± 0.01 (n = 14)] in MCT21/CLZ + were significantly lower than the values of the MCT21/CLZ- group (Fig. 5A, D, F), suggesting that CLZ treatment ameliorated the development of PH and RVH. No significant differences in mAP were observed among the 4 groups (mAP: Sal21/CLZ- 104.44 ± 3.59 (n = 9), Sal21/CLZ + 108.89 ± 10.03 (n = 9), MCT21/CLZ- 93.56 ± 1.80 (n = 16), MCT21/CLZ + 103.71 ± 6.92 (n = 14) mmHg) (Fig. 6A).

**Fig. 7:**
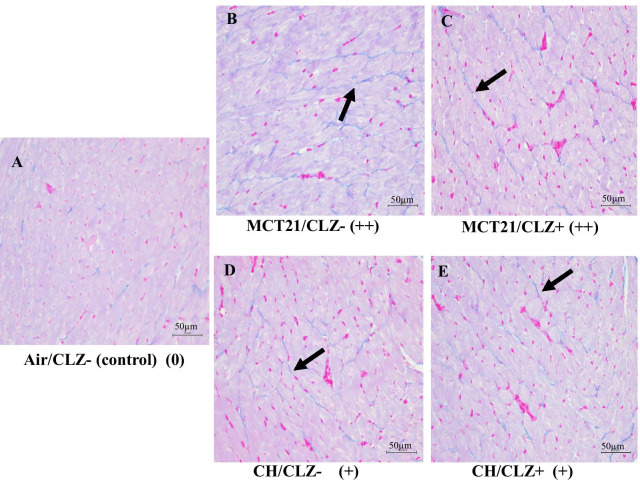
Collagen staining in the right ventricle. Representative photomicrographs of collagen staining in the right ventricle detected using Masson’s trichrome staining. Images were captured with a microscope at × 400 magnification, and the scale bars in lower right corner represent 50 µm. **A**: Air/CLZ- (control), rats exposed to ambient air and fed rat chow without CLZ; **B**: MCT21/CLZ-, rats injected with MCT and fed rat chow with CLZ; **C**: MCT21/CLZ +, rats injected with MCT and fed rat chow without CLZ; **D**: CH/CLZ-, rats exposed to chronic hypoxia and fed rat chow without CLZ; **E**: CH/CLZ +, rats exposed to chronic hypoxia and fed rat chow with CLZ. Fibrosis scale: 0 = normal, (+) = mild, and (++) = moderate. The blue color indicates collagen (allows)

The staining of collagen fibers in the right ventricle was obtained in control rat (Fig. 7A). The staining of collagen fibers in the right ventricle was increased in MCT 21 rat, but CLZ treatment did not seem to decrease this increased staining (Fig. 7B, C).

#### MCT28 model

**Fig. 8:**
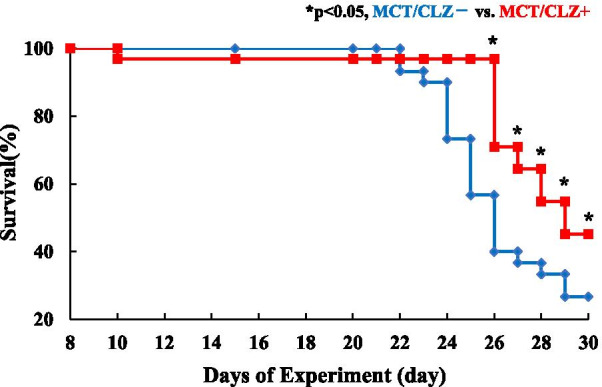
Kaplan–Meier survival curve of rats treated with and without CLZ after the MCT injection. Thirty rats treated with CLZ (MCT/CLZ +) (red line) and 30 rats treated without CLZ (MCT/CLZ-) (blue line) were used. MCT/CLZ-, rats injected with MCT and fed rat chow without CLZ; MCT/CLZ +, rats injected with MCT and fed rat chow with CLZ. *P* < 0.05 compared to rats treated without CLZ

PH in MCT rats is progressive, and PAP becomes extremely high. For MCT28 rats, the effect of CLZ was evaluated by measuring systolic right ventricular pressure (sRVP). We measured systolic right ventricular pressure (sRVP) in MCT28 rats under anesthesia instead of awake mPAP because obtaining mPAP in the MCT28 group is technically difficult. Anesthesia might affect sRVP. When comparing the effects of MCT administration between the Sal28/CLZ- and MCT28/CLZ- groups, sRVP and RV/LV + S were significantly higher in the MCT28/CLZ- group: sRVP, 30.20 ± 3.35 (n = 3) versus 76.63 ± 3.20 (n = 8) mmHg (*P* < 0.05) (Fig. 5C); RV/LV + S, 0.28 ± 0.02 (n = 5) versus 0.63 ± 0.04 (n = 8) (*P* < 0.05) (Fig. 5H). In the comparison of the effects of CLZ treatment between the MCT28/CLZ + and MCT28/CLZ- groups, sRVP [35.28 ± 1.90 (n = 8) mmHg (*P* < 0.05) (Fig. 5C)] and RV/LV + S [0.32 ± 0.04 (n = 9) (*P* < 0.05)] in the MCT28/CLZ + group were significantly lower than the values of the MCT28/CLZ- group (Fig. 5H), suggesting again that CLZ treatment ameliorated the development of PH and RVH. CLZ treatment significantly prolonged survival for an additional 4 days beginning at 26 days after the MCT injection in rats with MCT-induced PH (Fig. 8).

#### CH model

CH caused PH with an mPAP of 29.1 ± 1.5 mmHg (n = 10) (CH/CLZ-) compared to 17.5 ± 0.5 mmHg in the Air/CLZ- group (n = 10) (*P* < 0.05). No significant differences were observed between rats in the CH/CLZ + and CH/CLZ- groups (Fig. 5B). The RV/LV + S was higher in the CH/CLZ- group at 0.41 ± 0.03 (n = 13) than in the Air/CLZ- group at 0.27 ± 0.06 (n = 10) (*P* < 0.05). Significant differences in RV/LV + S were not observed between the CH/CLZ + and CH/CLZ- groups (Fig. 5G). The mPAP/mAP ratio was also higher in the CH/CLZ- group than in the Air/CLZ- group, with no significant difference between the CH/CLZ + and CH/CLZ- groups (Fig. 5E). No significant differences in mAP were observed among the 4 groups (Fig. 6B). (mAP: Air/CLZ- 105.0 ± 2.15 (n = 8), Air/CLZ + 101.88 ± 2.06 (n = 8), CH/CLZ- 104.0 ± 2.39 (n = 13), CH/CLZ + 103.78 ± 3.66 (n = 14) mmHg). The staining of collagen fibers in the right ventricle was obtained in control rat (Fig. 7A). Staining for collagen fibers in the right ventricle was increased in CH rat, but the CLZ treatment did not seem to decrease this increased staining (Fig. 7D, E).

### Vascular structural changes

#### MCT21 model

**Fig. 9:**
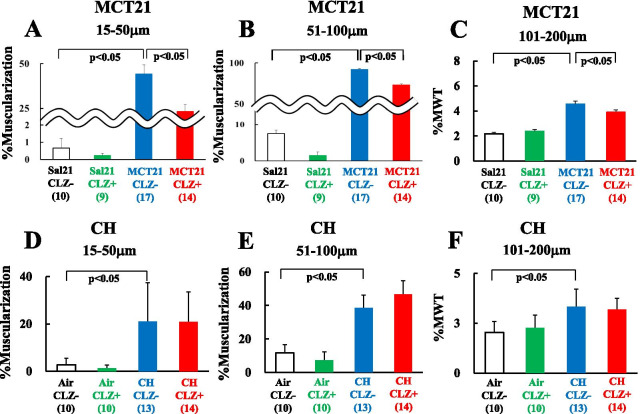
%Muscularization and %MWT in rats injected with monocrotaline (MCT) and exposed to chronic hypoxia (CH). The data were obtained from the MCT21 model (rats fed with and without CLZ for 21 days after the injection of saline or MCT) and CH model (rats fed with and without CLZ for 14 days during exposure to ambient air or chronic hypoxia). %Muscularization, the percentages of muscularized arteries in peripheral pulmonary arteries with an external diameter between 15 and 50 µm (**A** and **D**) and those with an external diameter between 51 and 100 µm (**B** and **E**). %MWT, %Medial wall thickness, is the ratio of the wall thickness of the media (distance between external and internal elastic laminae) to the external diameter in muscular arteries with diameters ranging from 101 to 200 µm (**C** and **F**). Sal21/CLZ-, rats injected with saline and fed rat chow without cilostazol (CLZ); Sal21/CLZ +, rats injected with saline and fed rat chow with CLZ; MCT21/CLZ-, rats injected with MCT and fed rat chow without CLZ; MCT21/CLZ +, rats injected with MCT and fed rat chow with CLZ; Air/CLZ-, rats exposed to ambient air and fed rat chow without CLZ; Air/CLZ +, rats exposed to ambient air and fed rat chow with CLZ; CH/CLZ-, rats exposed to chronic hypoxia and fed rat chow without CLZ; CH/CLZ +, rats exposed to chronic hypoxia and fed rat chow with CLZ. (n) = numbers of rats, means ± SE

The %Muscularization was higher in arteries with external diameters between 15 and 50 µm (Fig. 9A) and arteries with diameters between 51 and 100 µm (Fig. 9B) in the MCT21/CLZ- group (15–50 µm 44.25 ± 5.11%, 51–100 µm 92.24 ± 2.98%) (n = 17) than in the Sal21/CLZ- group (15–50 µm 0.66 ± 0.55% (*P* < 0.05), 51–100 µm 7.50 ± 3.43% (*P* < 0.05)) (n = 10). The MCT21/CLZ + group (15–50 µm 23.36 ± 3.73% (*P* < 0.05), 51–100 µm 73.46 ± 5.80% (*P* < 0.05)) (n = 14) exhibited a significantly lower %Muscularization in both sizes of arteries than the MCT21/CLZ- group (Fig. 9A, B). The %MWT in the MCT21/CLZ- group (4.56 ± 0.24% (n = 17)) was higher than that in the Sal21/CLZ- group (2.18 ± 0.13% (n = 10)) (*P* < 0.05) in the muscular arteries with external diameters ranging from 101 and 200 µm and usually accompanied by terminal or respiratory bronchioles (Fig. 9C). The MCT21/CLZ + group (3.91 ± 0.17% (n = 14) (*P* < 0.05)) had a significantly lower %MWT than the MCT21/CLZ- group (Fig. 9C).

#### CH model

The %Muscularization (Fig. 9D, E) and %MWT (Fig. 9F) were significantly higher in the CH/CLZ- group (%Muscularization: 15–50 µm 20.97 ± 4.37%, 51–100 µm 38.51 ± 7.79%; %MWT: 101–200 µm 3.32 ± 0.26%) (n = 13) than in the Air/CLZ- group (%Muscularization: 15–50 µm 2.74 ± 0.91% (*P* < 0.05), 51–100 µm 11.59 ± 5.03% (*P* < 0.05); %MWT: 101–200 µm 2.04 ± 0.18% (*P* < 0.05)) (n = 10), whereas the CH/CLZ + (%Muscularization: 15–50 µm 20.89 ± 3.50%, 51–100 µm 46.71 ± 8.02%; %MWT: 101–200 µm 3.19 ± 0.16%) (n = 14) was not significantly different from the CH/CLZ- group (Fig. 9D–F).

### Hematocrit

Hematocrit was similar among the Sal21/CLZ-, Sal21/CLZ +, MCT21/CLZ- and MCT21/CLZ + groups: 43.9 ± 0.7% (n = 9), 41.4 ± 1.4% (n = 9), 41.3 ± 1.5% (n = 16), and 42.4 ± 1.5% (n = 14), respectively. Hematocrit in the CH/CLZ- group (56.1 ± 1.6%, n = 14) was significantly higher than in the Air/CLZ- group (42.8 ± 1.7%, n = 9). The CH/CLZ + group (56.1 ± 1.6%, n = 15) had values similar to those of the CH/CLZ- group (Fig. 6C, D).

### Western blotting and PCR

#### eNOS and peNOS

**Fig. 10:**
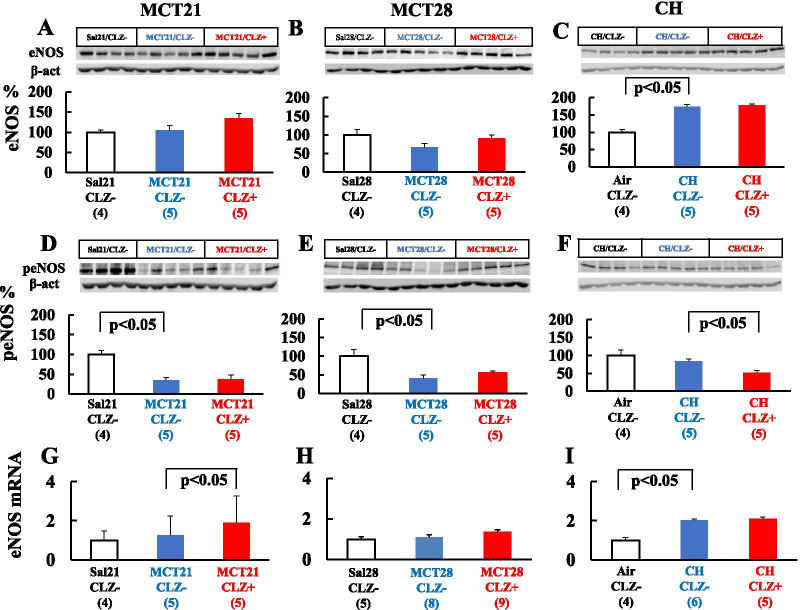
Levels of the endothelial nitric oxide synthase (eNOS) and phosphorylated eNOS proteins and eNOS mRNA. The left column (MCT21) represents rats in the MCT21 group.; The middle column (MCT28) represents rats in the MCT28 group.; The right column (CH) represents rats in the CH group. **A**: eNOS protein in the MCT21 group; **B**: eNOS protein in the MCT28 group; **C**: eNOS proein in the CH group; **D**: peNOS protein in the MCT21 group; **E**: peMOS protein in the MCT28 group; **F**: peNOS proein in the CH group; **G**: eNOS mRNA in the MCT21 group; **H**: eNOS mRNA in the MCT28 group; **I**: eNOS mRNA in the CH group. Representative Western blot images showing levels of the eNOS and peNOS proteins (**A**-**F)** are presented. Sal, injected with saline; MCT, injected with monocrotaline (MCT). Air, exposed to ambient air; CH, rats exposed to chronic hypoxia; CLZ-, fed rat chow without CLZ; CLZ +, fed rat chow with CLZ. The average intensity of the Sal/CLZ- group in the MCT21 and MCT28 models and Air/CLZ- group in the CH model was set to 100%. The intensity of each sample was calculated as a percentage of the average value (relative intensity). (n) = number of rats, means ± SE

MCT had no effect on eNOS protein expression (Fig. 10A, B), whereas CH upregulated eNOS protein expression (Fig. 10C). MCT also had no effect on eNOS mRNA levels (Fig. 10G, H), whereas CH increased eNOS mRNA expression (Fig. 10I). MCT decreased the level of the peNOS protein (Fig. 10D, E), whereas CH did not (Fig. 10F). CLZ increased eNOS mRNA levels in the MCT21 group (Fig. 10G) and decreased peNOS levels in the CH group (Fig. 10F).

#### AKT and pAKT

**Fig. 11:**
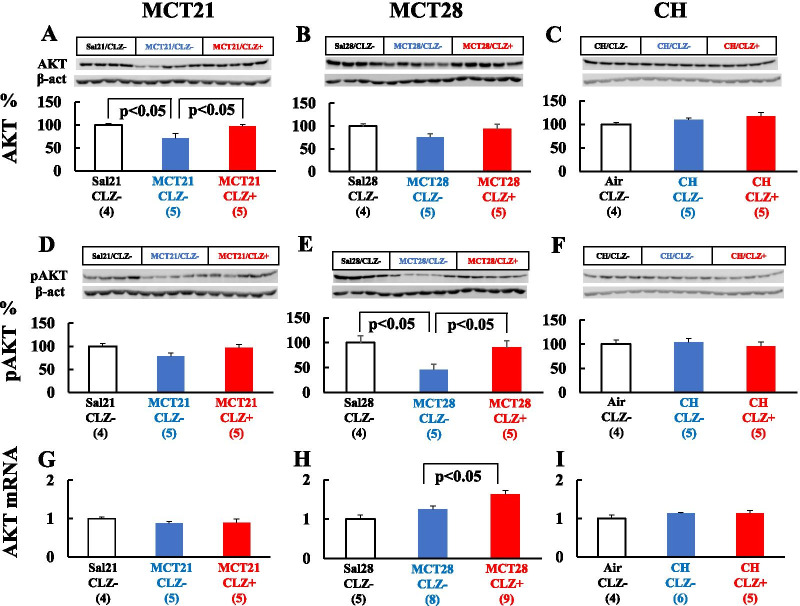
Levels of the AKT and phosphorylated AKT (pAKT) proteins and AKT mRNA. **A**: AKT protein in the MCT21 group; **B**: AKT protein in the MCT28 group; **C**: AKT protein in the CH group; **D**: pAKT protein in the MCT21 group; **E**: pAKT protein in the MCT28 group; **F**: pAKT proein in the CH group; **G**: AKT mRNA in the MCT21 group; **H**: AKT mRNA in the MCT28 group; **I**: AKT mRNA in the CH group. Representative Western blot images show AKT and pAKT protein levels (**A**-**F**). Sal, injected with saline; MCT, injected with monocrotaline (MCT). Air, exposed to ambient air; CH, rats exposed to chronic hypoxia; CLZ-, fed rat chow without CLZ; CLZ +, fed rat chow with CLZ. (n) = number of rats, means ± SE. See Fig. 9 for the definitions of abbreviations

MCT significantly downregulated the level of the AKT protein in the MCT21 model (Fig. 11A) and insignificantly downregulated in MCT28 model (Fig. 11B). MCT also downregulated the pAKT protein insignificantly in MCT21 model (Fig. 11D) and significantly in the MCT28 model (Fig. 11E). CLZ significantly attenuated this decrease in pAKT protein levels in the MCT28 model (Fig. 11E). Although CLZ did not increase the AKT mRNA levels in MCT21 model (Fig. 11G), CLZ slightly but significantly increased AKT mRNA expression in the MCT28 model (Fig. 11H). In the CH model, the levels of the AKT mRNA, AKT protein and pAKT protein were unchanged, and CLZ had no effect (Fig. 11C, F, I).

#### IκB and HMGB-1

**Fig. 12:**
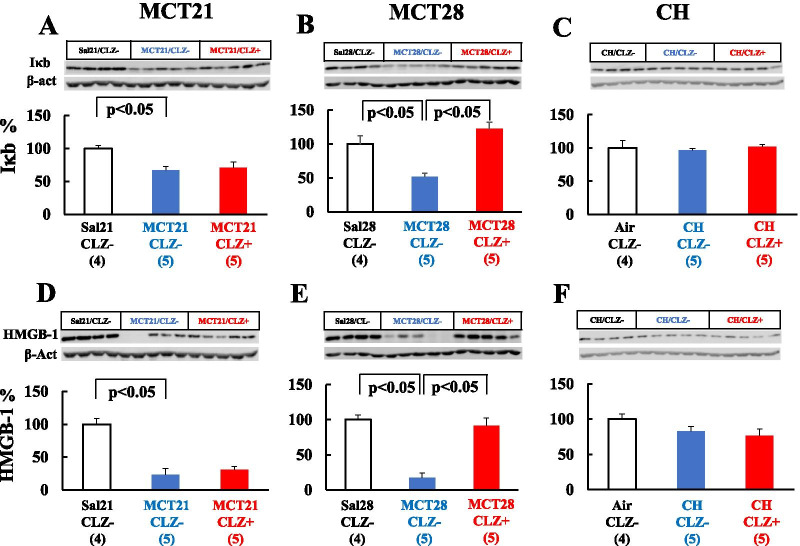
Western blot analysis of IκB and HMGB-1 levels. **A**: IκB protein in the MCT21 group; **B**: IκB protein in the MCT28 group; **C**: IκB proein in the CH group; **D**: HMGB-1 protein in the MCT21 group; **E**: HMGB-1 protein in the MCT28 group; **F**: HMGB-1 proein in the CH group. Representative Western blot images showing IκB and HMGB-1 levels (**A**–**F**). Sal, injected with saline; MCT, injected with monocrotaline (MCT). Air, exposed to ambient air; CH, rats exposed to chronic hypoxia; CLZ-, fed rat chow without CLZ; CLZ +, fed rat chow with CLZ. HMGB-1, High-mobility group box-1. (n) = number of rats, means ± SE. See Fig. 9 for the definitions of abbreviations

MCT downregulated IκB (Fig. 12A, B) and HMGB-1 (Fig. 12D, E) protein expression in both the MCT21 and MCT28 models (Fig. 12A,B, D, E). CLZ significantly attenuated the downregulation of both IκB (Fig. 12B) and HMGB-1 (Fig. 12E) protein expression in the MCT28 model. In the CH model, the expression of the IκB and HMGB-1 proteins was unchanged, and CLZ had no effect (Fig. 12C, F).

#### MCP-1

**Fig. 13:**
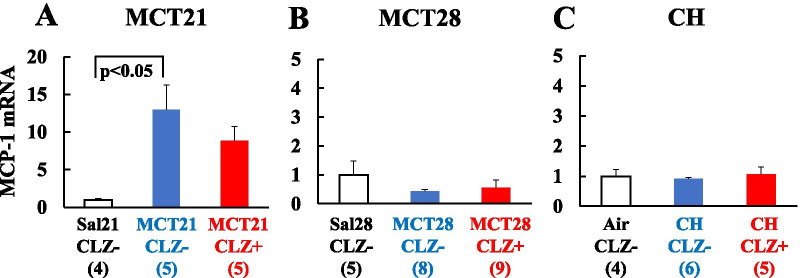
Expression of the MCP-1 mRNA. **A**: MCP-1 mRNA in the MCT21 group; **B**: MCP-1 mRNA in the MCT28 group; **C**: MCP-1 mRNA in the CH group. Sal, injected with saline; MCT, injected with monocrotaline (MCT). Air, exposed to ambient air; CH, rats exposed to chronic hypoxia; CLZ-, fed rat chow without CLZ; CLZ +, fed rat chow with CLZ. MCP-1, Monocyte chemotactic protein-1. (n) = number of rats, means ± SE. See Fig. 9 for the definitions of abbreviations

The MCP-1 mRNA was expressed at higher levels in the MCT21/CLZ- group than in the Sal21/CLZ- group. No significant difference was observed between the MCT21/CLZ- and MCT21/CLZ + groups (Fig. 13A). In the MCT28 and CH models, differences in MCP-1 mRNA levels were not observed among the groups (Fig. 13B, C).

## Discussion

The MCT and CH models were used to determine the difference in the effect of CLZ on the development of PH in these models. CLZ treatment ameliorated the development of PH and RVH and the increase in %Muscularization (percentages of muscularized arteries in peripheral pulmonary arteries) and %MWT (medial wall thickness of muscular arteries) in the MCT PH model. In contrast, CLZ did not ameliorate these hypertensive changes in the CH model. In the MCT-exposed lung, the IκB protein level decreased and the MCP-1 mRNA level increased, whereas these changes were not detected in the CH model.

This study had several limitations. First, the present results were only obtained from adult male rats, and we must be cautious when discussing neonatal and juvenile rats and/or infant and pediatric human patients, since age and sex influence PH caused by CH [53], and animal models do not completely recapitulate the human disease [16]. In most studies using animal models of PH, investigators have only used male rats [8, 10, 11, 13, 15, 43, 48, 53, 54]. Since we also only used male rats to establish rat models of experimental PH [6, 12, 31, 45], we used only male rats in the present study according to our own and other researchers’ studies. Second, a higher dosage of CLZ was administered in the present study than in the previous study [43]. We used this concentration because chow containing 0.3% CLZ was administered to spontaneously hypertensive rats [52]. The rats in the previous study [43] did not have lethal conditions compared with the rats in the present study. Third, the protocol was preventive rather than therapeutic after the occurrence of PH. However, an interventional treatment approach would represent a better translational approach, e.g., treatment starting on day 21 in the MCT model. Fourth, this study is observational and not mechanistic. Because ① CLZ increases AKT phosphorylation in cell-based studies [38, 40], ② PI3/AKT-dependent NO production has been reported [38, 55], and ③ NO production and/or administration ameliorates the development of hypertensive pulmonary vascular changes [6, 7, 33], we determined the levels of the AKT, pAKT, eNOS mRNA, eNOS, and peNOS proteins. Since an attenuation of the decrease in the levels of pAKT, an active form of AKT, was detected in the MCT model with no changes in the CH model, this effect of CLZ might partially explain the difference in the effect of CLZ on the two models. Although one of the targets of pAKT is eNOS [55], we did not detect an increase in peNOS levels in the whole lung. Immunohistochemistry of lung tissue might help identify cells with increased peNOS expression and further determine the relationship between pAKT and peNOS.

Although we did not measure cardiac output in the present study, another study showed no significant difference in cardiac output between saline control rats and MCT rats 2 and/or 3 weeks after the MCT injection [8, 13, 48]. Because systemic arterial pressure was similar among the four groups in the present study, we postulated that cardiac output had been maintained in our MCT21 group. However, as MCT rats ultimately develop right heart failure and potentially develop substantial myocardial inflammation, an assessment of right ventricular function (e.g., cardiac output) and right ventricular changes, such as apoptotic index, right ventricular fibrosis or changes in capillary density, might be helpful. Accordingly, we tried to evaluate fibrosis in the right ventricle by performing Masson’s trichrome staining for collagen in a pooled sample of the heart tissues that were originally used to determine RVH, and found that collagen fiber staining in the right ventricle was increased in both MCT and CH rats, but CLZ treatment did not seem to decrease these changes. Thus, we did not detect an effect of CLZ on fibrosis in hypertrophic right ventricles. Although technical issues might exist, we did not detect a decrease in right ventricle fibrosis based on collagen staining in CLZ-treated MCT rats, which might simply suggest that CLZ does not correct fibrosis. Combined with the result of another study using ultraviolet-irradiated mice showing that CLZ increased collagen staining in skin [56], CLZ did not decrease the collagen density.

A high hematocrit contributes to a high PAP in the CH model in addition to hypertensive pulmonary vascular remodeling [11], where the decrease in hematocrit is expected to reduce PAP. Since the hematocrit in both the CH and MCT models was not changed by CLZ, the attenuation of the mPAP by CLZ treatment was not due to the decrease in hematocrit. Medial hypertrophy of the muscular artery indicates the hypertrophy and hyperplasia of vascular smooth muscle cells, whereas new muscularization of normally nonmuscular arteries indicates the differentiation of pericytes to mature smooth muscle cells [11, 12]. Because CLZ induces NO production through NOS activation [37, 38] and an increase in endogenous NO levels might ameliorate the development of hypertensive pulmonary vascular changes [6, 33], we determined eNOS and peNOS levels in lungs from both models.

According to previous studies, the expression of the eNOS mRNA is increased in the lungs of CH-induced PH rats [51, 57], consistent with the present results. Since CLZ increases NOS expression in cultured endothelium [37, 38], we expected to observe the upregulation of NOS in lung tissue from the CH group exposed to CLZ. CLZ might have no effect on NOS synthesis, at least in whole lung tissue from CH-exposed rats, since eNOS protein levels in the lung tissue were similar between CH-induced PH rats treated with and without CLZ. Based on this result, the combined CH and CLZ treatment did not further increase eNOS expression compared with CH-induced NOS upregulation. Although eNOS mRNA expression was increased by CLZ in the MCT21 model, which is consistent with an earlier study [43], CLZ had no effect on the levels of the eNOS and peNOS proteins in either model. We are unable to easily explain the preventive effect of CLZ on the MCT model through increased NO production.

MCT-induced PH rats [6, 8, 12–15, 17, 18, 33, 43, 45–50] have been used to investigate pulmonary vascular remodeling in inflammatory-related PH, including ARDS [4, 5]. CH-induced PH [6, 7, 9–11, 31, 32, 51] is a type of PH caused by hypoxia and has been observed in patients residing at high altitude and patients with chronic obstructive pulmonary disease. Endothelial injury precedes the increase in PAP in MCT-induced PH [48], whereas the increase in PAP precedes the development of vascular changes in CH-induced PH [10]. A previous study showed that chronic NO inhalation prevented the development of PH and pulmonary vascular remodeling in CH-induced PH [7] but not in MCT-induced PH [12]. NO inhalation causes selective pulmonary vasodilation. Thus, the different effects of inhaled NO on different PH models suggest that reversing vasoconstriction is effective at preventing the development of some forms of PH in which vasoconstriction is the initial insult. Since CLZ did not prevent the development of CH-induced PH, we speculate that CLZ has a less potent pulmonary vasodilating effect. Endothelial injury is the initial insult in MCT-induced PH [48]. CLZ has been reported to promote endothelial regeneration in injured carotid arteries [58] and endothelial proliferation in lymphatics [54].

Because ① the expression of IκB is decreased in lungs from MCT-induced PH rats [45]; ② the increase in IκB levels induced by pyrrolidine dithiocarbamate (PDTC), an NFκB inhibitor, ameliorated the development of hypertensive pulmonary vascular changes [45]; and ③ CLZ reduces NFκB activity [59, 60], we determined the expression of IκB in lung tissue and the effect of CLZ. NFκB is a transcription factor that regulates the transcription of genes involved in inflammatory responses. A decrease in levels of the IκBα protein has been reported to be associated with increased NFκB activity [61]. IκBα expression was unchanged in CH-induced PH rats but decreased in MCT-induced PH rats in the current study, consistent with our previous study [45] suggesting that inflammatory components play a greater role in the etiology of MCT than in the CH model. Although we were unable to identify the IκB-expressing cells using Western blot analysis because we used whole lung tissue, endothelial cells and mononuclear cells might express this protein because a previous study in our lab showed an increased number of endothelial cells and infiltrated mononuclear cells that express the phosphorylated p65 subunit of NFκB in the nucleus [45]. The increased lung expression of the MCP-1 mRNA in the present study also supports the inflammatory component of the MCT model. The mechanism and role of MCP-1 in the inflammatory response are chemotactic and activating effects on monocytes/macrophages [50]. In the MCT model, we and others have documented macrophage infiltration into the alveolar wall by 14 days after MCT injection [15, 45]. The plasma and bronchoalveolar lavage fluid (BALF) MCP-1 levels increase transiently and then return to normal levels [50]. We also showed that lung expression of the MCP-1 mRNA was elevated in MCT21 rats in the present study and showed increases in BALF MCP-1 and TNFα levels measured using ELISAs in a previous study [14], suggesting the presence of inflammation in MCT rats. The anti-inflammatory effect of CLZ [40, 62] might explain the reversal of decreased levels of IκBα in the lungs of MCT-injected rats. CLZ has been reported to inactivate NFκB [59, 60]. Moreover, retinoic acid prevents the development of MCT-induced PH by inhibiting MMP-1 [47]. NFκB induces the expression of MMP-1 [63], and CLZ was reported to prevent MMP-1 activity in a cell-based study [64].

Because previous studies showed an increased plasma HMGB-1 level in subjects with both MCT- and chronic hypoxia-induced PH [46], we determined the expression of HMGB-1 in lung tissue and the effect of CLZ. HMGB-1 is normally present as a nuclear protein and is passively released from damaged cells [41]. The lower expression of HMGB-1 in the lung tissue of MCT-injected rats might be due to the increased release of HMGB-1 protein into the circulation, which might reflect damage to the cells. A recent study showed that an increase in serum HMGB-1 is associated with a concurrent decrease in tissue HMGB-1 protein expression [65]. CLZ restored HMGB-1 protein expression to the control level at 28 days after the MCT injection, potentially indicating the ability of CLZ to ameliorate cell damage and prolong survival. Furthermore, CLZ inhibits lipopolysaccharide-activated HMGB-1 release and prolongs the survival of endotoxemic mice [62].

The balance of pulmonary arterial smooth muscle cell proliferation and apoptosis is important to maintain structural and functional integrity. In the MCT model, increased pulmonary arterial smooth muscle cell proliferation and decreased apoptosis are pathogenic mechanisms. Decreased apoptosis might lead to the development of PH, whereas the induction of apoptosis relieves PH [2]. Since CLZ exerts an anti-apoptotic effect [43], the inhibitory effect of CLZ on the development of PH might not be due to the induction of apoptosis.

A decrease in IκB levels in the MCT model and no changes in IκB levels in the CH model revealed the etiological difference between these two models; MCT is an inflammatory stimulus, whereas CH is less inflammatory. We speculate that the anti-inflammatory effects of CLZ might have partially influenced the different physiological and pathological effects of CLZ on the two PH models investigated in the present study. CLZ might ameliorate inflammatory experimental PH. However, from a clinical perspective, the use of CLZ as a PH treatment might be limited. The effect of CLZ was partial, since the mPAP and RV/LV + S did not return to control levels, because the %Muscularization and %MWT were only partly ameliorated. The currently registered drugs for PH, such as prostacyclin and/or its analogs, endothelin-receptor blockers, and PDE5 inhibitors, ameliorate the development of PH in both MCT and CH models [17–20]. We expected that CLZ would prevent PH in both models, but it did not.

## Conclusions

The administration of CLZ prevented the development of PH in a rat model of MCT-induced PH, although CH-induced PH development was not prevented by CLZ. The inhibitory effect of CLZ on the development of PH might depend on the etiology of PH, and alterations in lung AKT, pAKT, and IκB levels might partially be related to its effects.

## Data Availability

All data generated or analyzed during this study are included in this published article [and its supplementary information files].

## Abbreviations

PH: Pulmonary hypertension
CLZ: Cilostazol
MCT: Monocrotaline
CH: Chronic (hypobaric) hypoxia
RV/LV + S: The right ventricle/ weight-to-left ventricle + septum weight ratio
mPAP: Mean pulmonary artery pressure
mAP: Mean artery pressure
sRVP: Systolic right ventricular pressure
PCR: Polymerase chain reaction
%Muscularization: Percentages of muscularized arteries
%MWT: Percent medial wall thickness
eNOS: Endothelial nitric oxide synthase
AKT: Serine-threonine protein kinase
MCP-1: Monocyte chemotactic protein-1
